# Gas Plasma Treatment Improves Titanium Dental Implant Osseointegration—A Preclinical In Vivo Experimental Study

**DOI:** 10.3390/bioengineering10101181

**Published:** 2023-10-11

**Authors:** Myron Nevins, Chia-Yu Chen, Stephano Parma-Benfenati, David M. Kim

**Affiliations:** 1 Department of Oral Medicine, Infection and Immunity, Division of Periodontology, Harvard School of Dental Medicine, Boston, MA 02115, USA; nevinsperimp@aol.com (M.N.); chia-yu_chen@hsdm.harvard.edu (C.-Y.C.); 2 Independent Researcher, Corso della Giovecca, 155, 44121 Ferrara, Italy; info@studioparmabenfenati.it

**Keywords:** plasma treatment, dental implant, osseointegration, bone-to-implant contact, sandblasted large grit acid-etched surface, histology, histomorphometic analysis

## Abstract

Recent technological advancements led to the development of various plasma-based technologies for post-packaging modifications. The purpose of the present preclinical in vivo study was to assess the safety and efficacy of a novel chairside nonthermal gas plasma treatment for enhancing osseointegration of titanium implants. Six male mixed foxhounds underwent extraction of mandibular premolars and first molars, and the sockets healed for 42 days. Canine mandibles were randomized to receive either plasma-treated (test) or non-plasma-treated (control) dental implants. A total of 36 implants were placed in six animals, and they were sacrificed at 2 weeks (two animals), 4 weeks (two animals), and 6 weeks (two animals) after the implant surgery. When the radiographic analysis was performed, the changes in bone level were not statistically significant between the two groups at 2 weeks and 4 weeks. The difference became significant at 6 weeks (*p* = 0.016), indicating more bone loss from baseline to 6 weeks for the control group. The bone-to-implant contact (BIC) appeared to be higher for the test groups at all time points, and the BIC was significantly higher for the test group at 4 weeks (*p* = 0.046). In conclusion, this study underscored the potential of nonthermal plasma treatment in enhancing implant osseointegration.

## 1. Introduction

Titanium dental implants are considered one of the most preferred and predictable treatment options for patients with missing teeth and occlusal rehabilitation [1]. The short- and long-term success of titanium dental implants is attributed to their ability to establish osseointegration, defined as the direct structural and functional connection between the living bone and the surface of a load-bearing implant [2]. A myriad of factors, such as implant design, surface composition, surface chemistry, surface roughness, surgical techniques, and the host–immune response, play a significant role in whether the titanium implant will achieve osseointegration or fibrointegration [3,4]. The dental implant’s primary stability depends on the shape and surface morphology, and the secondary stability depends mainly on the implant surface [4].

The implant surface characteristics, particularly surface roughness and hydrophilicity, are regarded as one of the most critical factors in achieving a high bone-to-implant contact (BIC) [5,6]. With enhanced surface energy and wettability, a hydrophilic surface has been reported to promote superior bone wound healing and osseointegration [7]. However, recent studies have indicated that titanium implants, from the time they are manufactured and packaged, undergo an aging process characterized by the accumulation of hydrocarbon contamination on the surface, which adversely affects surface hydrophilicity and suppresses cell recruitment and biological activities [8,9,10,11]. This time-dependent degradation could potentially lead to decreased osseointegration and affect the survival of the dental implants.

Current alternatives to minimize or remove hydrocarbon contaminations include the storage of implants in saline solution and using UV lights or argon plasma to remove organic compounds and modify the surfaces to become hydrophilic. Ultraviolet (UV) light irradiation technology, first introduced in 1997, has been employed to rejuvenate aged or contaminated implant surfaces by creating hydrophilic surfaces, a process facilitated by the removal of water molecules and partial elimination of hydrocarbons [12]. Several studies reported accelerated implant stability, higher BIC, and a reduced clinical implant healing time [12,13,14,15,16]. UV treatment also decreased both carbon impurities on the surface and water contact angles [16]. However, Mehl reported no significant effects on osseointegration of dental implants after UV photofunctionalization [17]. In addition, the combination of variables such as duration, intensity, and wavelength can create various modes of UV photofunctionalization, and the cost of the technology might be hampering its routine use in dental practice [18,19].

To address these limitations of post-implant packaging sterilization methods, recent technological advancements led to the development of various plasma-based technologies for post-packaging modifications [20,21,22,23,24,25]. Nonthermal plasma treatment via dielectric barrier discharge (DBD) is a technique used for surface modification. Previous studies showed that plasma treatment significantly reduces hydrocarbon impurities and increases osseointegration efficacy [19,20]. Low-temperature plasma can be used to “prime” or “improve” surfaces to turn them medically biocompatible, such as functionalization of implant surfaces [19]. The treatment should be performed immediately prior to the implant placement to yield the most clinical benefits, since the atmospheric hydrocarbons readily adsorb to the surface, thereby reducing hydrophilicity.

The purpose of the present preclinical in vivo study was to assess the safety and efficacy of a novel chairside nonthermal gas plasma treatment (ACTILINK reborn, Plasmapp Co. Ltd., Daejeon, Republic of Korea) in enhancing osseointegration of titanium implants. This is a cleaning system that uses the Bio-RAP™ process to generate plasma on the implant surface while in a vacuum state. The use of plasma under these conditions reduces hydrocarbon impurity buildup on the implant’s surface. Through clinical, radiographic, and histologic evaluations, we aim to investigate the effect of plasma treatment on osseointegration and bridge the gap in knowledge concerning real-time surface modification of titanium implants to set the groundwork for future clinical applications.

## 2. Materials and Methods

### 2.1. Ethics Statement

The selection and management of experimental animals and the research protocol were approved by the Institutional Animal Care and Use Committee (IACUC), Pine Acres Rabbitry/Farm, Norton, MA, USA (Approval #22-05). The ARRIVE (Animal Research: Reporting of In Vivo Experiments) guidelines were followed for reporting the findings.

### 2.2. Sample Size Calculation

Based on the difference in bone-to-implant contact (BIC) from previous studies [26,27], it was estimated that at least 5 implants per group per time point would be needed by power calculation under the significance of 5% and the power of 80%. To account for unexpected events, 6 implants were allocated per group per time point. Thus, to compare plasma-treated implants versus untreated controls at three time points, 36 implants were necessary. With up to 3 implants per jaw or 6 implants per animal, 6 animals were required for the study.

### 2.3. Housing, Husbandry of the Experimental Animals

Six healthy male mixed foxhounds aged over 1 year, weighing 25 kg and above, were prepared for this split-mouth-designed study. The animals were properly nurtured and given a suitable diet under regular laboratory settings, and they were accommodated in an environment with a room temperature between 15 to 20 degrees Celsius and a humidity level exceeding 30%.

### 2.4. Treatment Group Allocation and Experimental Materials

Based on a split-mouth design, each side of the posterior mandible of each animal was randomly assigned to one of the following two groups:

- Control group: non-plasma-treated dental implants, n = 18 implants; 6 implants biopsied at 2 weeks, 6 implants biopsied at 4 weeks, and 6 implants biopsied at 6 weeks.
- Test group: plasma-treated dental implants, n = 18 implants; 6 implants biopsied at 2 weeks, 6 implants biopsied at 4 weeks, and 6 implants biopsied at 6 weeks.

### 2.5. Surgical Procedures

#### 2.5.1. Tooth Extractions

General anesthesia using Aceapromazine 0.005–0.5 mg/kg, max dose of 2 mg, and Telazol 1–4 mg/kg was administered, followed by inhalation of 1.5 to 2% isoflurane for the duration of the procedure. Local anesthesia (2 carpules of 2% Xylocaine with 1:100 K epi, local infiltration, 3.6 mL) was administered intraorally. Clinical pictures were taken before the start of the surgery. Sulcular incisions were made around premolars (P1–P4) and first molar (M1) with #15 blade, and the subsequent reflection of full mucoperiosteal flaps was performed using an elevator. Following thorough plaque and calculus removal with dental scalers, P2-M1 were hemisected and both mesial and distal roots of each tooth were extracted. The surgical site was closed with resorbable sutures (3-0 Vicryl Rapide sutures) using the interrupted suturing technique. Clinical pictures were taken at the end. The animals underwent the standard post-surgical infection and pain control (cefazolin sodium (20 mg/kg, i.m) for a minimum of 3 days and buprenorphine HCL (0.02 mg/kg, i.m. BID or as needed) for a minimum of 5 days). The animals received a diet of softened food (Purina Pro Plan dog food) soaked for 45 min in warm water during the entire healing period and treatment phase. When animals came off the surgical table, they were transported out of the surgical suite and placed on a heating pad where their vitals (heart rate, breathing rate, SPO2, and reflex responses) were checked every 15 min until the animals could maintain sternal recumbence on their own. The animals were then transported back to their holding pens.

#### 2.5.2. Dental Implant Placement

**Figure 1:**
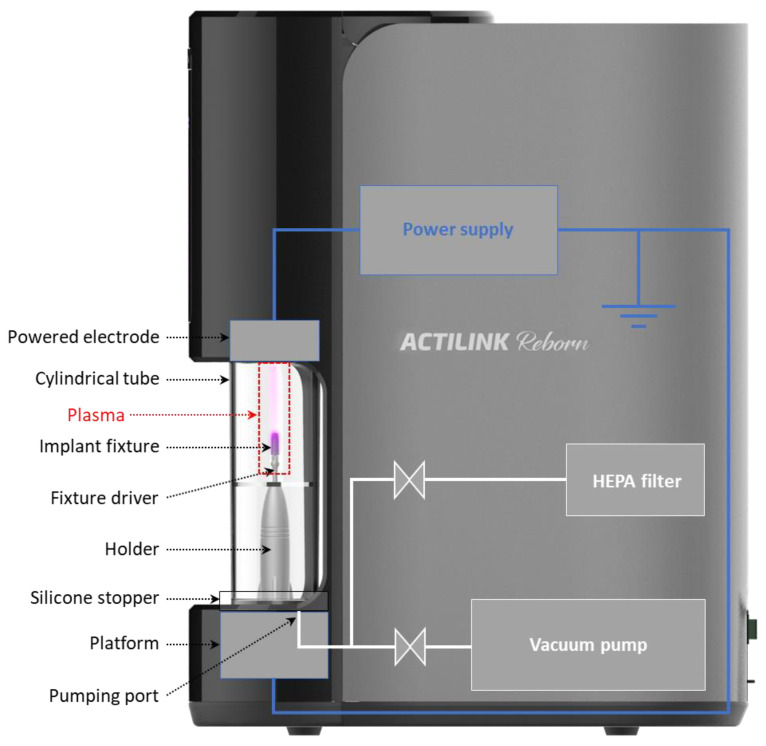
Dental implant undergoing plasma treatment before the implant insertion.

After a healing period of 42 days, the same surgical protocol was utilized to place six implants into each animal (three implants on each side) according to a randomized distribution pattern generated for each animal before the surgery. Clinical pictures were taken before the start of the surgery. General anesthesia using Aceapromazine 0.005–0.5 mg/kg, max dose of 2 mg, and Telazol 1–4 mg/kg was administered, followed by inhalation of 1.5 to 2% isoflurane for the duration of the procedure. Local anesthesia (2 carpules of 2% Xylocaine with 1:100 K epi, local infiltration, 3.6 mL) was administered intraorally. Implant osteotomies were performed with torque reduction rotary instruments at 1000 rpm using a sterile saline solution. Each animal’s mandibular jaw was randomized to receive either test (plasma-treated) or control (non-plasma-treated) dental implants (SF 4.0 × 8.5 mm, Hoowon EDI, grade 4 titanium with 0.5% Fe, 0.08% C, 0.4% O, 0.05% N, and 0.015% H). For the test group, dental implants were plasma-treated by placing dental implants into an ACTILINK Reborn (Plasmapp Co., Ltd. Daejeon, Republic of Korea) device to undergo 1 min of plasma treatment cycle immediately before implant insertion (Figure 1). The ACTILINK Reborn uses a fixture driver, which the dental implant is loaded onto, that is seated into a holder. Once the holder is properly positioned on the platform, the chamber is lowered until it contacts the silicon cover embedded in the platform’s floor. The seal created via the chamber and silicon cover allows for a partial vacuum to be formed via the vacuum port connected to the vacuum pump.

As depicted in Figure 1, the plasma treatment process involved installing a fixture driver and loading the dental implant on a sterile holder dedicated to the ACTILINK Reborn device. Once the holder was properly positioned in the ACTILINK Reborn, a cylindrical Pyrex^®^ tube was lowered and engaged with the silicone stopper below, separating the implant and the ambient air. The engagement between the cylindrical tube and silicone stopper formed an airtight seal, allowing a vacuum condition inside the tube through the vacuum port connected to the vacuum pump. This resulted in a base pressure of approximately 5 torr within 30 s. The dental implant was electrically connected to a grounding electrode of the device to discharge the plasma onto the implant surface. Once applying sinusoidal electric power with a frequency of 100 kHz and a voltage of 3 kV to the powered electrode placed on the top of the cylindrical tube, plasma was generated and treated on the implant surface for 8 s. To remove the impurities detached from the implant surface through the vacuum port and enhance the purification performance, the pumping continued for an additional 17 s after the plasma treatment. The final step of the plasma treatment process involved venting the vacuum chamber for approximately 5 s. The gas inlet was connected to the HEPA filter to eliminate the possible re-attachment of impurities in ambient air. Therefore, the total plasma treatment cycle consisted of vacuum generation (30 s), plasma treatment (8 s), purification with a by-product elimination phase (17 s), and venting (5 s).

**Figure 2:**
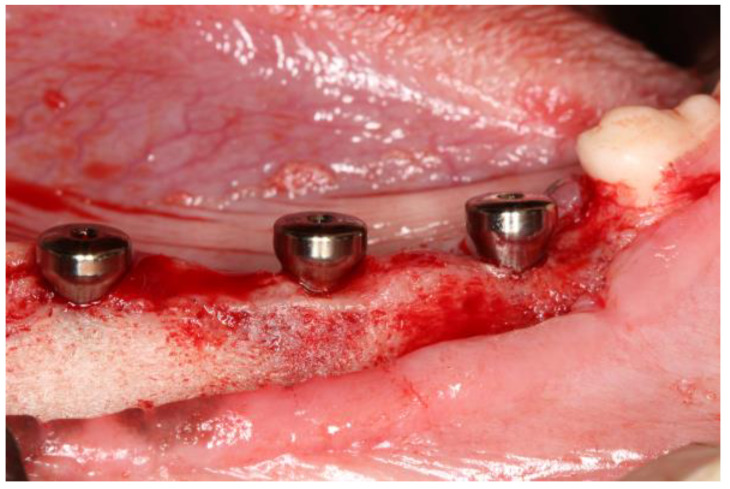
Clinical picture of test group at 4 weeks after connecting healing abutments to dental implants.

Dental implants were placed with an implant manufacturer-recommended insertion device (Insertion torque value or ITV measured) and a hand rachet according to the manufacturer’s guidelines. Prior to healing abutment insertion, implant stability quotient (ISQ) values were measured with ISQ measuring device (Osstell Beacon, Osstell, Göteborg, Sweden) (Figure 2).

Clinical pictures were taken before the flap adaptation. The flaps were adapted around healing abutments (4 mm diameter and 5 mm height, Hoowon EDI for tension-free wound closure with resorbable interrupted sutures (3-0 Vicryl Rapide Sutures)). The animals underwent the standard post-surgical infection and pain control (cefazolin sodium (20 mg/kg, i.m) for a minimum of 3 days and buprenorphine HCL (0.02 mg/kg, i.m. BID or as needed) for a minimum of 5 days). The animals received a diet of softened food (Purina Pro Plan dog food) soaked for 45 min in warm water during the entire healing period and treatment phase. When animals came off the surgical table, they were transported out of the surgical suite and placed on a heating pad where their vitals (heart rate, breathing rate, SPO2, and reflex responses) were checked every 15 min until they could maintain sternal recumbence. The animals were then transported back to their holding pens.

#### 2.5.3. Sacrifice of the Experimental Animals

The animals were sacrificed at 2 weeks (2 animals), 4 weeks (2 animals), and 6 weeks (2 animals) after the implant surgery. Euthanasia was performed using Aceapromazine 0.005–0.5 mg/kg, max dose of 2 mg, Telazol 1–4 mg/kg, and Euthasol (10 mg/lb IV). The mandibles were resected en bloc using an oscillating autopsy saw, and the recovered specimens were immediately immersed in fixative for histological preparation and evaluation.

### 2.6. Radiographic Assessment

**Figure 3:**
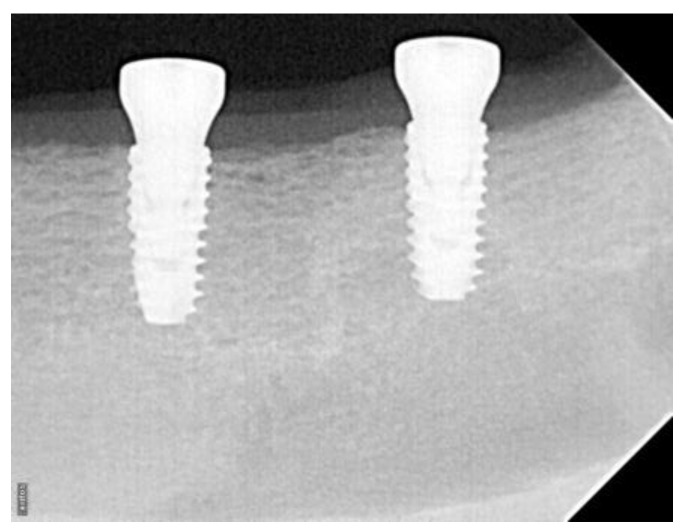
The radiograph of a 6-week test group implant revealing stability of bone level around dental implant threads.

Standardized periapical dental radiographs were taken at the time of the implant placement and at the time of the sacrifice (2 weeks, 4 weeks, and 6 weeks) (Figure 3).

The radiographs were saved in JPEG format. The images were standardized based on known implant diameter and length and digitally measured using ImageJ software (National Institutes of Health, Bethesda, MD, USA). A built-in digital caliper in the software was used for all measurements, and pixel values of a given linear measurement were converted to millimeters. The implant platform and the first radiographic implant–bone contact were identified on the mesial and distal surfaces. Baseline and follow-up radiographs were used to calculate the changes in the level of first implant–bone contact.

### 2.7. Descriptive Histology and Histomorphometric Analysis

The fixed samples were dehydrated in a graded series of ethanol (60%, 80%, 96%, and absolute ethanol) using a dehydration system with agitation and vacuum. The blocks were infiltrated with Kulzer Technovit 7200 VLC-resin. Infiltrated specimens were placed into embedding molds, and polymerization was performed under blue- and white light. Polymerized blocks were sectioned in a mesio-distal direction and parallel to the long axis of each implant. The slices were reduced by microgrinding and polishing using an Exakt grinding unit to an even thickness of 60–70 µm. Sections were stained with RBS and counter-stained with acid fuchsin and examined using both a Leica MZ16 stereomicroscope and a Leica 6000DRB light microscope. Histomorphometric measurements were performed by using software (ImageAcess, Imagic, Switzerland) to calculate the percentage of osteoid, mineralized new bone, and remaining old bone along the bone–implant contact surface. BIC was calculated based on the sum of osteoid, mineralized new bone, and remaining old bone.

### 2.8. Scanning Electron Microscopy (SEM) with Energy Dispersive X-ray Spectroscopy (EDS) and X-ray Photoelectron Spectroscopy (XPS)

To compare the amount of impurity or the level of carbon content before and after plasma treatment, the implant was examined by SEM (Hitachi, SU5000) with EDS (Hitachi, SU5000) and XPS (ULVAC-PHI, PHI Quantera II) before and after the plasma treatment. The imaging aimed to analyze the changes in carbon levels, so the site where the impurity existed was enlarged and imaged. After the plasma treatment, the exact same position was imaged.

### 2.9. Statistical Analysis

The data were represented by mean ± SD and analyzed statistically using SPSS software (version 23.0, IBM, Armonk, NY, USA). Parametric and non-parametric pairwise comparisons with Student *t*-test and Wilcoxon signed-rank test were conducted between the control and plasma-treated groups. The criterion for statistical significance was set at *p* < 0.05.

## 3. Results

### 3.1. SEM, EDS and XPS Surface Analysis

**Figure 4:**
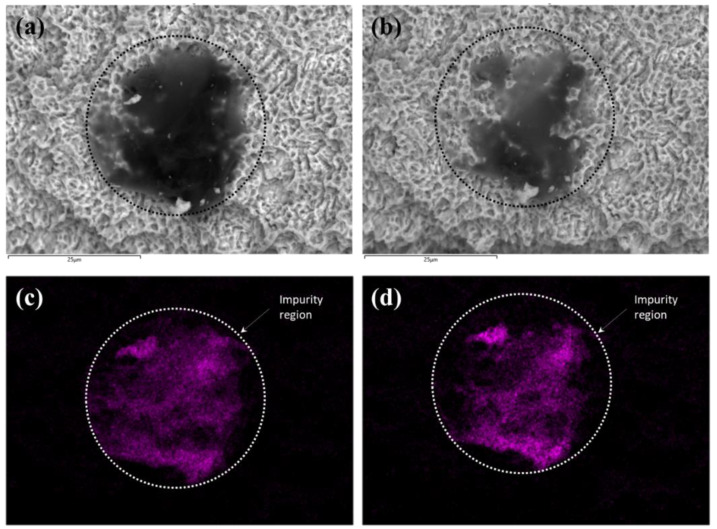
Representative SEM images from (**a**) control and (**b**) test implant and corresponding EDS images showing carbon distribution of (**c**) control and (**d**) test implant. Black circle in (**a**,**b**), and white circle in (**c**,**d**) indicate the region containing a microsized impurity.

**Figure 5:**
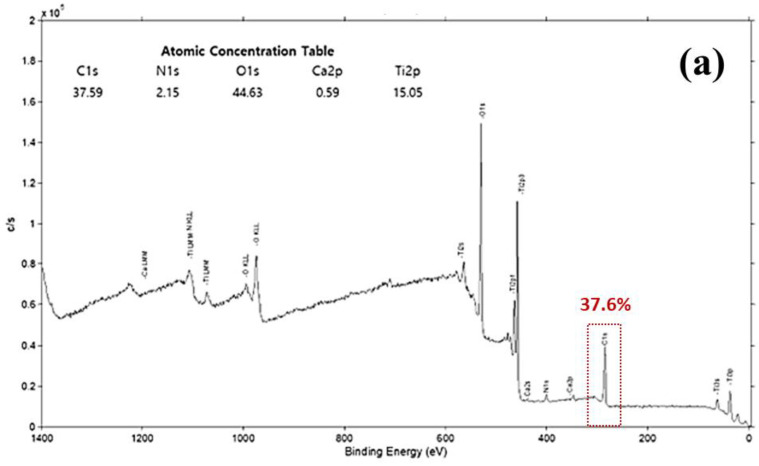

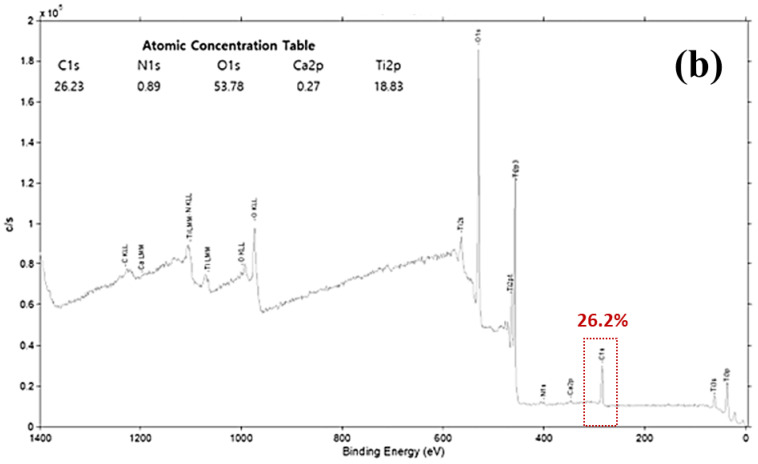
XPS spectra before (**a**) and after (**b**) plasma treatment.

The SEM and EDS images shown in Figure 4 revealed that the size of carbon impurities was reduced by plasma treatment. The black shaded areas in Figure 4a,b appear to show non-metallic substances, and these were confirmed to be carbon-based impurities by the EDS image shown in Figure 4c,d, which visualize the distribution of carbon and show a thick accumulation within the circle. The atom content measured in the control and plasma-treated implant surfaces changed from 11.07% to 7.63%, indicating a 31% reduction of carbon impurity through plasma treatment. Additionally, the XPS spectra revealed that the C1s peak decreased by plasma treatment from about 38% to about 26%, while Ti2p and O1s peaks increased by removing the carbon impurities (Figure 5).

### 3.2. Clinical Findings

**Figure 6:**
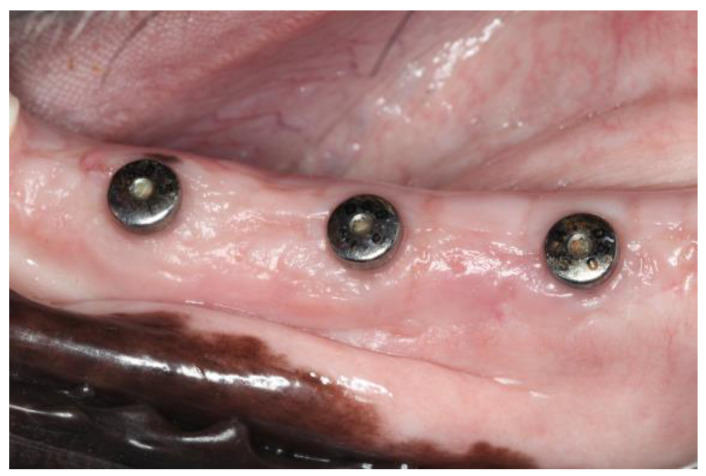
Clinical picture of 4-week control implants demonstrating healthy soft tissue support around healing abutments.

A total of 36 implants were placed in six animals. Clinically, all implants seemed stable except one control implant from the 2-week group that lost the healing abutment (Figure 6).

### 3.3. ITV, ISQ and Radiographic Analysis

**Figure 7:**
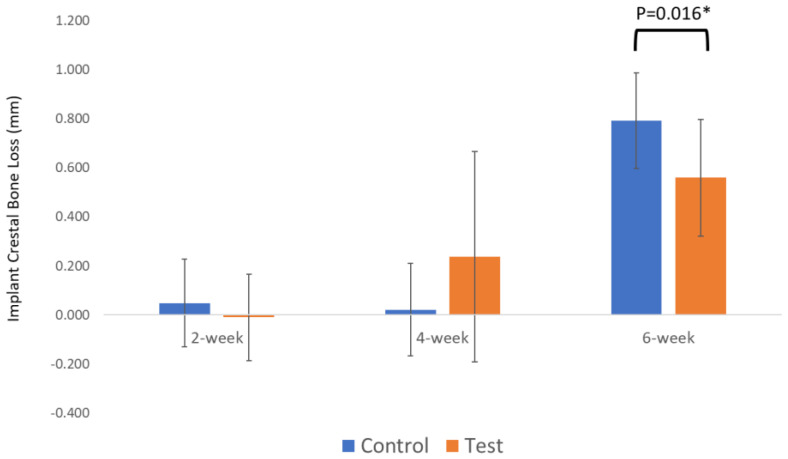
Periapical radiograph analysis revealing statically significant differences in bone level between control and plasma implants at week 6 (* symbolizes statistical significance).

All implants reached an ITV of 40 Ncm, and some implants had to be hand-torqued. At baseline, there was no statistical difference between the groups for ISQ (79.39 ± 2.95 for control and 79.53 ± 4.05 for test, *p* = 0.6). On radiographic analysis, the implants were placed subcrestally in both groups with no statistical significance. When the radiographic analysis was performed, the changes in bone level were not statistically significant between the two groups at 2 weeks (*p* = 0.44) and 4 weeks (*p* = 0.13). The difference became significant at 6 weeks (0.79 mm ± 0.20 for the control and 0.56 mm ± 0.24 for the test, *p* = 0.016), indicating more bone loss from baseline to 6 weeks for the control group (Figure 7).

### 3.4. Histomorphometric Analysis and Bone-to-Implant Contact (BIC) Analysis

Histology results revealed three dental implants that failed to osseointegrate (one test implant from 2 weeks and two control implants from 6 weeks), and the histomorphometric analyses were performed excluding three failed dental implants. For 2-week specimens, de novo bone formation was observed along the implant threads. For 4- and 6-week specimens, bone formation continued to occur in all specimens, primarily observed by increased bone volume fill inside and around the implant threads. There were no statistical differences for the areas of osteoid, new bone, and old bone between the control and plasma-treated groups at all time points.

**Figure 8:**
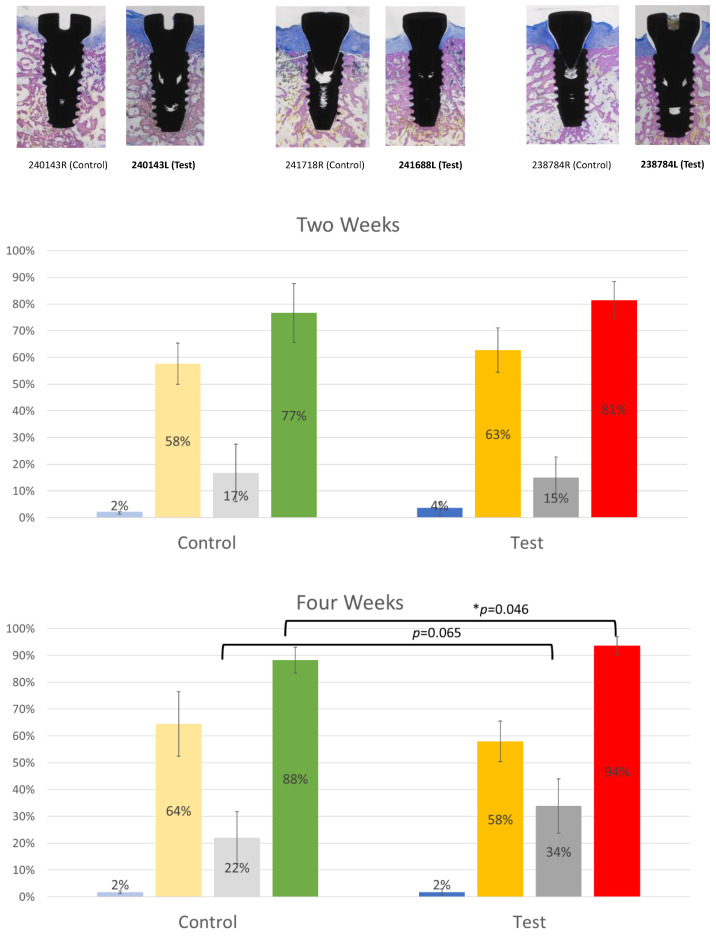

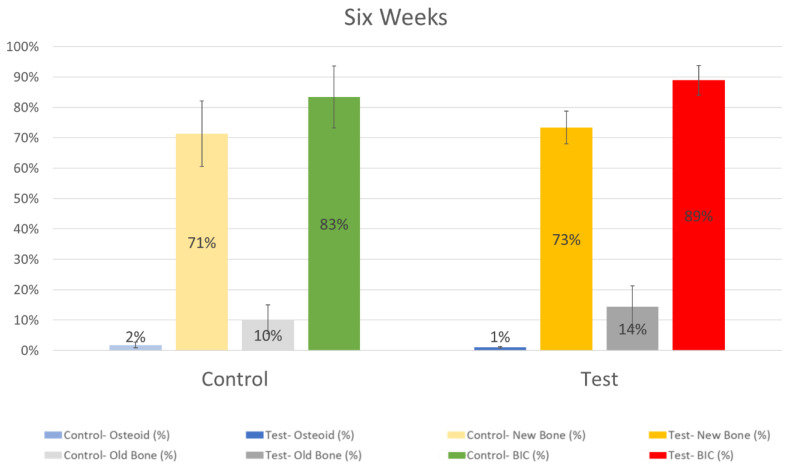
Representative histology images from control and test implants for each evaluation time point (2 weeks, 4 weeks, and 6 weeks). Histomorphometric analysis examined osteoid %, new bone %, old bone %, and BIC % (* symbolizes statistical significance).

The BIC appeared to be higher for the plasma-treated groups at all time points, and the BIC was significantly higher for the plasma-treated group at 4 weeks (88.3 ± 4.8% versus 93.7 ± 3.3%, *p* = 0.046) (Table 1, Figure 8).

## 4. Discussion

Dental implant surface properties, such as chemical composition, electrical charge, roughness, surface energy, morphology, and wettability, play an important role in determining the cascade of biological events that allow osseointegration [4]. Dental implant surface treatments, such as plasma spray, laser treatment, acid etching, anodizing, nanoparticle depositions, and sand blasting, followed by acid etching have all allowed for faster osseointegration and reduction in loading time [4].

The concept of biological aging of dental implants prior to implant placement and after implant placement has been a topic of interest for many years [9]. However, it is important to understand that an earlier implant aging process may begin immediately after the manufacturing, sterilization, and packing of dental implants. Most dental implants have a shelf life of 5 years, and dental surgeons may not obtain dental implants until at least 6 months after packaging. The surface aging process due to carbon contamination is inevitable because the sterilized implants are exposed to the ambient air before being inserted into the patient [19]. Impurities such as hydrocarbon contaminants can cause adverse effects on cellular adhesion and protein adsorption, resulting in early marginal bone loss [19].

The present study aimed to investigate the effects of nonthermal plasma treatment on dental implants for osseointegration in a canine model. The implant utilized for this study had a surface roughness of 1.3~1.8 μm with a sandblast large grit acid-etched surface. In clinical applications, various methods, including insertion torque and resonance frequency analysis, are typically utilized to quantify primary stability. Secondary stability, or osseointegration, is usually gauged by the overall outcome measure of implant survival. In research, measures such as resonance frequency analysis (RFA), mechanical disruption testing, and histomorphometric analysis are often utilized to gain additional insights into the rate and extent of osseointegration.

The results showed that plasma treatment did not significantly affect the implant torque value (ITV) and implant stability quotient (ISQ) at the time of implant insertion. We decided not to measure ISQ during the observation period in order to avoid disrupting the osseointegration process. The radiographic bone level during the early osseointegration stages (2 and 4 weeks) was comparable; however, at the 6-week mark, the plasma-treated group showed significantly higher radiographic bone levels than the non-plasma-treated control group.

Histology and histomorphometric analysis were needed to confirm these findings. The percentage of osteoid, new bone, old bone, and BIC were all assessed around the study implants. For all evaluation points, there was a trend of higher BIC for the plasma-treated group, but it was statistically significant only at the 4-week evaluation point. This observation is in line with other surface functionalization techniques, such as UV functionalization. In a UV study conducted in rats, significant results for bone–implant integration assessed via push-in test were observed at both 2 and 4 weeks [12]. Given the faster healing rate in rats compared to canines, our results at week 4 are consistent with the expected timeline of osseointegration in the canine model. One point of interest is the lack of significant difference in BIC at week 6. However, it is crucial to highlight the secondary observation of increased crestal bone loss in the control group by this time. The greater bone loss in the untreated implants suggested a more extended advantage of plasma treatments.

Our results further substantiate findings from previous in vitro studies that have reported the beneficial effects of plasma treatment on dental implants. Duske et al. reported that cold atmospheric plasma treatment reduced contact angle and supported the spreading of osteoblastic cells; furthermore, the treatment effectively removed the biofilm [28,29]. Berger et al. demonstrated that a benchtop plasma treatment at the time of implant placement could alter the surface energy of an implant without modifying the chemical composition and enhance osteoblast cell differentiation [20]. Lee et al. have conducted extensive in vitro studies, such as hydrocarbon contamination analysis, protein adsorption assay, cell proliferation assay, cell differentiation assay, and scanning electron microscope (SEM) analysis, to examine the effect of plasma treatment on dental implants [19]. With SEM analysis, there was no noticeable difference between the conditions of the sandblast large grit acid-etched surface before and after the plasma treatment in terms of cracking or corrosion sites. Their study revealed a 58% reduction of hydrocarbons, a 25% increase in protein adsorption, a 39% increase in cell attachment to the implant surface, and an 82% increase in alkaline phosphatase activity. Their results indicate that plasma treatment efficiently eliminates the hydrocarbon, enhancing protein adsorption and improving cell adhesion, proliferation, and differentiation.

The ACTIINK Reborn utilized in this study consists of a vacuum pump and high-voltage power supply that discharges cylindrical plasma on the surface of the implant fixture. According to Lee et al., impurities including hydrocarbon can be effectively dissociated by ionized particles and radicals in plasma [19]. The threshold energy for hydrocarbon dissociation is approximately 20 eV, and the optimized vacuum pressure for removing hydrocarbon from the implant surface is numerically and experimentally found to be 5–10 torr [19]. The ACTILINK Reborn used in this study is designed to obtain a base pressure of 5 torr and an operating pressure in the range of optimized pressure conditions for activating the implant surface. Also, there is a large pressure gradient formed inside the tube by the vacuum pump, and we believe that this can facilitate the removal of the impurities from the implant surface and the tube through the vacuum port. Our SEM and EDS results confirmed the reduction of carbon impurities after plasma treatment (Figure 4).

It is worth noting that the dental implant surface (sandblast large grit acid-etched) that was used in this study is considered the gold standard in dental implantology. The fact that plasma treatment was able to enhance further the already favorable results observed in the control group underscores the potential of this technology in dental implantology. To the best of our knowledge, this is the first in vivo study investigating the effects of nonthermal plasma treatment on dental implants.

Our study, however, is not without limitations. The small sample size may limit the generalizability of our findings. Furthermore, we could not control occlusal forces and oral hygiene in the canines, possibly leading to the loss of three implants, potentially influencing the outcomes. Future research should aim to address these limitations and further investigate the long-term effects of plasma treatment on dental implants. Forthcoming clinical trials may provide more definitive evidence of the benefits of plasma treatment. Additionally, research into optimizing the plasma treatment protocol could further enhance its effectiveness.

## 5. Conclusions

In conclusion, this preclinical study underscored the potential of nonthermal plasma treatment in enhancing dental implant osseointegration. Despite a small sample size, the plasma-treated implants demonstrated superior osseointegration and reduced vertical bone loss, suggesting the potential for shorter healing times before proceeding with prosthetic loading and improved long-term stability. While further research is needed to validate and optimize this treatment, these findings highlight its promising clinical significance in potentially improving patient outcomes in dental implant therapy.

## Figures and Tables

**Table 1:** Statistical analysis demonstrating a significant difference in BIC between control and test implants at week 4.

	Control	Test	
	(Mean ± SD)	(Mean ± SD)	*p*-Value
Osteoid (%)			
2 weeks	2.2 ± 1.0%	3.6 ± 0.5%	0.366
4 weeks	1.7 ± 0.5%	1.8 ± 1.1%	0.853
6 weeks	1.8 ± 1.0%	1.1± 0.2%	0.224
New bone (%)			
2 weeks	57.7 ± 7.7%	62.8 ± 8.3%	0.316
4 weeks	64.4 ± 12%	58.0 ± 7.6%	0.292
6 weeks	71.4 ± 10.1%	73.4 ± 5.3%	0.914
Old bone (%)			
2 weeks	16.8 ± 10.8%	15.0 ± 7.7%	0.765
4 weeks	22.1 ± 9.7%	33.9 ± 10.1%	0.065
6 weeks	10.3 ± 4.7%	14.4 ± 6.8%	0.326
BIC (%)			
2 weeks	76.7 ± 11.0%	81.4 ± 6.9%	0.428
4 weeks	88.3 ± 4.8%	93.7 ± 3.3%	**0.046**
6 weeks	83.5 ±10.2%	88.9 ± 4.8%	0.284

## Data Availability

All data has been presented in the manuscript.
